# Mentoring in general surgery in Switzerland

**DOI:** 10.3402/meo.v20.27528

**Published:** 2015-03-31

**Authors:** Reto M. Kaderli, Jennifer M. Klasen, Adrian P. Businger

**Affiliations:** 1 Department of Visceral Surgery and Medicine, Bern University Hospital, University of Bern, Bern, Switzerland; 2 Department of Surgery, Kantonsspital Frauenfeld, Spital Thurgau AG, Frauenfeld, Switzerland; 3 Federal Department of Defense, Swiss Armed Forces, Bern, Switzerland; 4 Private University in the Principality of Liechtenstein, Triesen, Liechtenstein

**Keywords:** mentoring, general surgery, career advancement, Switzerland, survey

## Abstract

**Background:**

Mentorship has been found as a key factor for a successful and satisfying career in academic medicine and surgery. The present study was conducted to describe the current situation of mentoring in the surgical community in Switzerland and to evaluate sex differences regarding the impact of mentoring on career success and professional satisfaction.

**Methods:**

The study was designed as an anonymous national survey to all members of the Swiss Surgical Society in 2011 (820 ordinary and 49 junior members). It was a 25-item questionnaire addressing mentor–mentee relationships and their impact on the professional front.

**Results:**

Of the 869 mailed surveys, 512 responses were received (response rate: 58.9%). Mentor–mentee relationships were reported by 344 respondents (68.1%) and structured mentoring programs were noted in 23 respondents (6.7%). Compared to individuals without mentors, male mentees exhibited significantly higher subjective career advancement (5.4±1.2 vs. 5.0±1.3; *p*=0.03) and career development (3.3±1.9 vs. 2.5±1.7; *p*<0.01) scores, but the differences for female mentees were not statistically significant (4.7±1.1 vs. 4.3±1.2, *p*=0.16; 2.5±1.6 vs. 1.9±1.4, *p*=0.26; respectively). The pursuit of an academic career was not influenced by the presence of a mentor–mentee relationship for female (*p*=0.14) or male participants (*p*=0.22).

**Conclusions:**

Mentor–mentee relationships are important for the career advancement of male surgeons. The reason for the lack of an impact on the careers of female surgeons is difficult to ascertain. However, mentoring also provides lifelong learning and personal development. Thus, specific attention should be paid to the development of more structured mentoring programs for both sexes.

The term mentor can be traced to the friend of Odysseus, Mentor, in Homer's epic, ‘The Odyssey’ (1). Mentor was entrusted to care for Odysseus’ son, Telemachus, in Odysseus’ absence, and Mentor was asked to support Telemachus as a wise counselor and tutor. Following the historical meaning, mentorship is currently characterized as the provision of support from a senior person to a junior person to promote the professional and personal development of the less experienced trainee (2). Over the past 30 years, the beneficial effects of mentoring have been examined in various professional fields, such as management in business economics (3, 4). Mentor–mentee relationships in management are an important tool to promote careers for both parties (5). Two facets of career success can be differentiated, and both facets are positively associated with mentoring (6): objective career success, which is measured by financial and professional status, and subjective career success, which is associated with personal career goals and aspirations (7, 8).

Studies investigating careers in academic medicine have confirmed the key role of mentoring (6, 9). Mentoring improves social networks and vocational satisfaction (10, 11), increases productivity in terms of publications and successful grant proposals (9, 12), and enhances career satisfaction (9).

Both women and men benefit from having senior mentors (9). In academic medicine, the difficulties women experience finding a mentor and the traditional sex distribution have been discussed as the main impediments to the progression of their surgical careers (12, 13). Seven years after graduation, 50% of physicians in Switzerland have a mentor, but there is a substantial mentorship gap between women and men (40.7% of women versus 60.4% of men have a mentor) (6).

The role of the mentor's sex in terms of support for career advancement and personal advice is a controversial topic (9, 14, 15). However, in academic surgery, there is a shortage of same-sex mentors and role models for women (11, 13).

In a previous study, we assessed the value of mentor–mentee relationships for female surgeons in Switzerland (15). The present study was conducted not only to describe the current situation of mentoring in the entire surgical community of Switzerland but also to evaluate sex differences regarding the impact of mentoring on career success and professional satisfaction. The aim of the study was to lay the foundation for establishing effective mentoring programs in surgery.

## Methods

This study is based on an anonymous national survey of all members of the Swiss Surgical Society working in Switzerland. Members were identified using the freely accessible database of the Swiss Surgical Society (in 2011, there were 820 regular and 49 junior members living in Switzerland, including 111 women) (16). Data were collected in the summer of 2011. Response enhancement techniques included advance notification and mailed reminders. After reviewing an outline of the investigation, the research and ethical committee of Berne, Switzerland, determined that the survey did not require ethical approval. The data were collected, stored, analyzed, and shared in strict adherence with the ethical standards of our institution. To ensure participant anonymity, data from the participant questionnaires were entered into an anonymous database.

### Survey instrument

In addition to sociodemographic data, respondents were asked about the presence of mentor–mentee relationships (yes/no), the number of mentors that they have had previously, the mentors’ sexes and hierarchical positions, and the existence of structured mentoring programs (yes/no). Career advancement and career satisfaction were evaluated subjectively on a 7-point Likert scale that ranged from 1 (strongly disagree) to 7 (strongly agree).

In a 16-item portion of the questionnaire, the respondents’ perceived support for career advancement was evaluated based on the ‘Mentoring Function Items’ of Noe and the ‘Career Support Scale’ of Riley and Wrench (17, 18). The questions were subdivided into five scales: ‘networking’, ‘career planning’, ‘coaching’, ‘emotional support’, and ‘role model’ (Table 1). The ‘mentoring experience scale’ consists of the networking and career planning scales. Each item was rated on a 5-point Likert scale that ranged from 1 (strongly disagree) to 5 (strongly agree). The participants were divided into two subgroups based on the presence of a mentor.

**Table 1:** Questionnaire to evaluate subjective support for career advancement for the respondents (17, 18)

Characteristic
**Networking scale** a
There are individuals in my professional environment who …
1. promote contact with important superiors.
2. promote contact with individuals who have a positive effect on my career.
3. help me prepare for a promotion.
4. help me promote my career using their influence.
**Career planning scale** a
There are individuals in my professional environment who …
5. help me with career planning.
6. provide tips for my professional future.
7. encourage me to take charge of my surgical career.
**Coaching scale**
There are individuals in my professional environment who …
8. help me learn the technical aspects of my work.
9. often provide good technical advice.
**Emotional support scale**
There are individuals in my professional environment who …
10. listen to me when I talk about my concerns and feelings.
11. support me emotionally and encourage me during stressful times.
12. take a real interest in my personal advancement.
13. are kind to me.
**Role model scale**
There are individuals in my professional environment who …
14. I emulate with regard to surgical techniques and strategies.
15. are role models to me.
16. have qualities that I would like to adopt for myself.

a The ‘mentoring experience scale’ consists of the ‘networking scale’ and ‘career planning scale’.

The participants’ professional career development was assessed based on the following objective criteria (19–22): ‘talks at scientific conferences’, ‘number of publications’, ‘participation in research projects’, ‘full-time research activities’, ‘scholarships awarded’, ‘third-party funds awarded’, and ‘research awards obtained’. Based on a validated score from Buddeberg et al. (23), these items were summarized to obtain a comparable value for career success that ranged from 0 to 11.

### Statistical analysis

Continuous data were expressed as medians (range) or means (standard deviation (SD)), and dichotomous data were expressed as frequencies and percentages. The Mann–Whitney *U* test was used to compare continuous variables. Dichotomous and categorical outcomes were analyzed using Fisher's exact test. A logistic regression multivariate analysis was performed to evaluate the effect of having a mentor. All data were analyzed using SPSS version 13.0 (SPSS; Chicago, Illinois, USA). All statistical tests were two-sided with a significance level of 0.05.

## Results

Of the 869 mailed surveys, 512 responses were received (response rate: 58.9%). Replies were received from 448 men (87.5%) and 64 women (12.5%). Table 2 presents the participants’ characteristics.

**Table 2:** Participants’ characteristics by sex

Characteristic	Overall (*n*=512)	Women (*n*=64)	Men (*n*=448)	*p*-value
Age, median (range), y	50 (28–71)	43 (29–63)	50 (28–71)	<0.01
Professional status, no. (%)				0.41
Surgeon-in-training	22 (4.3)	4 (6.3)	18 (4.0)	
Board-certified surgeon	490 (95.7)	60 (93.8)	430 (96.0)	
Hierarchical position, no. (%)				<0.01
Resident	22 (4.3)	3 (4.7)	19 (4.2)	
Attending	109 (21.3)	32 (50.0)	77 (17.2)	
Consultant	94 (18.4)	9 (14.1)	85 (19.0)	
Head of department	123 (24.0)	5 (7.8)	118 (26.3)	
Physician in private practice	143 (27.9)	13 (20.3)	130 (29.0)	
Other	21 (4.1)	2 (3.1)	19 (4.2)	
Hospital category, no. (%)^a^ (2 missing values)				0.09
Type U	71 (13.9)	7 (11.1)	64 (14.3)	
Type A	116 (22.7)	18 (28.6)	98 (21.9)	
Type B3	44 (8.6)	4 (6.3)	40 (8.9)	
Type B2	67 (13.1)	8 (12.7)	59 (13.2)	
Type B1	41 (8.0)	10 (15.9)	31 (6.9)	
Private practice	97 (18.9)	6 (9.5)	91 (20.4)	
Other	74 (14.5)	10 (15.9)	64 (14.3)	
Married or with a partner, no. (%)	422 (82.4)	49 (76.6)	373 (83.3)	0.42
At least one child, no. (%) (1 missing value)	406 (79.3)	27 (42.2)	379 (84.8)	<0.01

a Type U: university hospitals, Type A: large referral centers, Type B3: regional or specialized hospitals, Type B2/B1: small regional surgical departments (classified according to the FMH) (24).

### Mentor–mentee relationships

A mentor–mentee relationship was experienced by 344/505 (68.1%) respondents (7 missing values; no significant difference was revealed regarding the frequency of mentor–mentee relationships between female and male participants (38/63 (60.3%) vs. 306/442 (69.2%), respectively; *p*=0.16). Table 3 presents the mentors’ characteristics.

**Table 3:** Characteristics of mentor–mentee relationships by participants’ sex

Characteristic	Overall (*n*=344)	Women (*n*=38)	Men (*n*=306)	*p*-value
Age of mentor, median (range), y	55 (35–70)	50 (35–65)	55 (38–70)	<0.01
Mentors’ sex, no. (%) (1 missing value)				<0.01
Female	6 (1.7)	5 (13.2)	1 (0.3)	
Male	337 (98.3)	33 (86.8)	304 (99.7)	
Mentors’ hierarchical position, no. (%) (1 missing value)				<0.01
Head of department at a university hospital	153 (44.6)	8 (21.1)	145 (47.5)	
Head of department at a non-university hospital	127 (37.0)	15 (39.5)	112 (36.7)	
Specialist registrar	55 (16.0)	12 (31.6)	43 (14.1)	
Specialist in private practice	6 (1.7)	2 (5.3)	4 (1.3)	
Other	2 (0.6)	1 (2.6)	1 (0.3)	
Structured mentoring program, no. (%) (2 missing values)	23 (6.7)	3 (7.9)	20 (6.6)	0.76
Number of mentors (to date), mean (SD)	1.7 (1.7)	1.4 (1.7)	1.8 (1.7)	0.41

The mentor's sex (female vs. male mentor) did not significantly affect the ‘mentoring experience scale’ of female (3.0±1.7 vs. 2.7±1.1; *p*=0.71) or male participants (2.3± (not available) vs. 2.7±1.3; *p*=0.73). A structured mentoring program did not have a significant impact on the ‘mentoring experience scale’ for female participants compared with a non-structured program (3.1±0.6 vs. 2.7±1.2, respectively; *p*=0.53); however, a significant increase in the mentoring experience was noted for structured programs for male participants (3.5±1.3 vs. 2.6±1.2; *p*=0.01).

### Impact of mentor–mentee relationships on professional careers

Mentor–mentee relationships did not have a significant effect on career satisfaction for female (5.5±1.3 with mentor vs. 5.5±1.1 without mentor; *p*=1.00) or male participants (5.7±1.2 with mentor vs. 5.5±1.3 without mentor; *p*=0.14).

An evaluation of the subjective support for career advancement based on the 16-item questionnaire in Table 1 did not reveal a significant impact of mentoring for female participants, whereas a significant impact was noted for male participants (Table 4). Similarly, mentor–mentee relationships did not have a significant impact on the subjective career advancement of female participants (4.7±1.1 with mentor vs. 4.3±1.2 without mentor; *p*=0.16). In contrast, male participants with mentors exhibited significantly increased subjective career advancement (5.4±1.2 vs. 5.0±1.3; *p*=0.03).

**Table 4:** Subjective support for career advancement depending on the presence of a mentor

	Overall *n*=64	With mentor *n*=38	Without mentor *n*=25				
					
Female participants	Mean (SD)	Mean (SD)	Mean (SD)	*p*-value	Shift	Lower CI	Upper CI
Networking scale	2.7 (1.1)	2.8 (1.2)	2.5 (1.0)	0.41	0.3	−0.3	0.8
Career planning scale	2.6 (1.3)	2.7 (1.3)	2.4 (1.2)	0.35	0.3	−0.3	1.0
Coaching scale	3.2 (1.1)	3.1 (1.2)	3.4 (0.8)	0.26	−0.5	−1.0	0.5
Emotional support scale	3.0 (1.0)	3.2 (0.9)	2.6 (1.2)	0.06	0.8	−0.0	1.3
Role model scale	3.0 (1.0)	3.2 (1.0)	2.8 (1.0)	0.26	0.3	−0.3	1.0
	Overall *n*=448	With mentor *n*=306	Without mentor *n*=136				
					
Male participants	Mean (SD)	Mean (SD)	Mean (SD)	*p*-value	Shift	Lower CI	Upper CI

Networking scale	2.5 (1.3)	2.7 (1.3)	2.1 (1.1)	<0.01	0.8	0.3	1.0
Career planning scale	2.4 (1.3)	2.6 (1.3)	2.0 (1.1)	<0.01	0.7	0.3	1.0
Coaching scale	3.0 (1.3)	3.1 (1.3)	2.7 (1.2)	<0.01	0.5	0.0	1.0
Emotional support scale	2.8 (1.1)	2.9 (1.1)	2.4 (1.0)	<0.01	0.5	0.3	0.8
Role model scale	2.8 (1.2)	3.0 (1.1)	2.5 (1.2)	<0.01	0.7	0.3	0.7

A similar difference for female and male participants was noted with regard to the objective criteria for professional career advancement. Mentoring did not cause an improvement for female participants, whereas male participants with mentors reported significantly more ‘talks at scientific conferences’, ‘participation in research projects’, ‘full-time research activities’, ‘scholarships awarded’, ‘third-party funds awarded’, and ‘research awards obtained’ (Table 5).

**Table 5:** Objective factors for career development depending on the presence of a mentor

			Overall *n*=64	With mentor *n*=38	Without mentor *n*=25				
							
Female participants		Item value for the career development score	*n* (%)	*n* (%)	*n* (%)	*p*-value	OR	Lower CI	Upper CI
Talks at scientific conferences	No talk	0	6 (10)	3 (8)	3 (15)	0.55			
	1–3 talks	1	18 (31)	11 (29)	7 (35)				
	≥4 talks	2	34 (59)	24 (63)	10 (50)				
Number of publications in peer-reviewed journals	No publication	0	18 (31)	11 (29)	7 (35)	0.70			
	1 publication	1	4 (7)	2 (5)	2 (10)				
	2–3 publications	2	16 (28)	10 (26)	6 (30)				
	≥4 publications	3	20 (34)	15 (39)	5 (25)				
Participation in research projects	Yes	1	23 (38)	16 (42)	7 (30)	0.42	1.6	0.5	5.9
	No	0	38 (62)	22 (58)	16 (70)				
Full-time research activities	None	0	57 (93)	35 (92)	22 (96)	0.72			
	≤9 months	1	1 (2)	1 (3)	0 (0)				
	>9 months	2	3 (5)	2 (5)	1 (4)				
Scholarships awarded	Yes	1	5 (8)	4 (11)	1 (4)	0.64	2.6	0.2	133.1
	No	0	56 (92)	34 (89)	22 (96)				
Third-party funds awarded	Yes	1	6 (10)	5 (13)	1 (4)	0.39	3.3	0.3	164.6
	No	0	55 (90)	33 (87)	22 (96)				
Research awards obtained	Yes	1	5 (8)	3 (8)	2 (9)	1.00	0.9	0.1	11.6
	No	0	56 (92)	35 (92)	21 (91)				
			Overall *n*=448	With mentor *n*=306	Without mentor *n*=136				
							
Male participants		Item value for the career development score	*n* (%)	*n* (%)	*n* (%)	*p*-value	OR	Lower CI	Upper CI

Talks at scientific conferences	No talk	0	35 (8)	16 (6)	19 (15)	0.01			
	1–3 talks	1	45 (11)	32 (11)	13 (10)				
	≥4 talks	2	333 (81)	240 (83)	93 (74)				
Number of publications in peer-reviewed journals	No publication	0	62 (15)	36 (12)	26 (20)	0.17			
	1 publication	1	22 (5)	14 (5)	8 (6)				
	2–3 publications	2	67 (16)	47 (16)	20 (16)				
	≥4 publications	3	266 (64)	192 (66)	74 (58)				
Participation in research projects	Yes	1	217 (50)	164 (54)	53 (40)	0.01	1.8	1.1	2.7
	No	0	218 (50)	139 (46)	79 (60)				
Full-time research activities	None	0	366 (85)	246 (82)	120 (92)	0.02			
			Overall *n*=448	With mentor *n*=306	Without mentor *n*=136				
Male participants		Item value for the career development score	*n* (%)	*n* (%)	*n* (%)	*p*-value	OR	Lower CI	Upper CI
	≤9 months	1	15 (3)	14 (5)	1 (1)				
	>9 months	2	51 (12)	41 (14)	10 (8)				
Scholarships awarded	Yes	1	67 (15)	55 (18)	12 (9)	0.02	2.2	1.1	4.7
	No	0	368 (85)	248 (82)	120 (91)				
Third-party funds awarded	Yes	1	125 (29)	101 (33)	24 (18)	<0.01	2.2	1.3	3.9
	No	0	310 (71)	202 (67)	108 (82)				
Research awards obtained	Yes	1	97 (22)	81 (27)	16 (12)	<0.01	2.6	1.4	5.1
	No	0	338 (78)	222 (73)	116 (88)				

For female participants, mentoring did not significantly impact the career development score (2.5±1.6 with mentor vs. 1.9±1.4 without mentor; *p*=0.26). However, the career development score of male participants was significantly increased in the presence of a mentor (3.3±1.9 vs. 2.5±1.7; *p*<0.01).

The pursuit of an academic career was not significantly influenced by the presence of a mentor–mentee relationship for female (4/36 (11%) with mentor vs. 0/24 (0%) without mentor; *p*=0.14) or male participants (24/292 (8%) with mentor vs. 6/134 (4%) without mentor; *p*=0.22) (26 missing values).

## Discussion

Whereas more than half of the members of the surgical community in Switzerland had a mentor, only a small proportion of participants reported involvement in a structured mentoring program. Mentoring exhibited a significant impact on subjective support for career advancement, subjective career advancement, and objective criteria for professional career advancement for male surgeons but not for their female counterparts. The pursuit of an academic career was not influenced by the presence of a mentor–mentee relationship.

Despite the commonly described lack of suitable mentors and limited amount of time (25), we found that 68% of Swiss surgeons of both sexes experienced a mentor–mentee relationship, which is consistent with a previous study among female surgeons in Switzerland (15). However, the percentage is higher than data from physicians in the United States (54–59%) or surgeons in the United Kingdom (49%) (26–28). Although a systematic review revealed more difficulties for women finding mentors compared with their male colleagues, we found similar percentages between both sexes (12). In the present study, each mentee had a mean of 1.7 mentors, which has been described as an asset in several publications. Different mentors may be advantageous for different aspects of the career (i.e., one may provide career guidance and support, another provides guidance on research, and a third junior mentor provides support for administrative tasks) (29, 30). This applies in particular to the fact that a single mentor is unlikely to provide all of the valuable characteristics of being an outstanding teacher, clinician, and researcher (2). The use of multiple mentors allows a single mentor to focus on his or her own expertise (31).

To make mentoring more accessible and to improve mentee satisfaction, the implementation of formal mentoring programs has been suggested (25, 32). We found that male surgeons with a structured program reported significantly better networking and career planning compared with males involved in a non-structured mentoring program. Nevertheless, structured mentoring programs were only experienced by 6.7% of respondents. Mentoring programs for medical students are more common in US medical schools than in Europe (33).

Female participants had a female mentor significantly more often than male participants. However, for both female and male participants, the mentor's sex did not significantly affect networking or career planning. Conflicting results have been reported in the literature on the impact of the mentor's sex on the effectiveness of counseling women about career advancement (34, 35). For example, male mentors might assume that women will not succeed in academic careers and therefore direct their attention elsewhere (36).

In a previous study, career progression and research were reported as the two most important areas of mentoring (28). Mentoring conveys an important role in research development as well as research productivity (37). Research has been defined as the most relevant factor for pursuing a prestigious career in medicine (23). The objective criteria for professional career advancement and the career development score of Buddeberg et al. were established based on this perceived importance of research (23). In the present study, an evaluation of subjective support for career advancement and career advancement itself exhibited a significant impact for male surgeons but not for their female counterparts. The same result was noted for the objective criteria for professional career advancement, with a significantly increased career development score for male participants only. Mentoring has been judged as being important for preventing participants, especially women, from abandoning their initial interest in academic careers (38, 39). The lack of an impact on objective criteria for professional career advancement can be potentially attributed to preexisting lower interest levels among female surgeons in academic pursuits (40).

According to the impact of mentoring on career progression and the research described above, mentoring has been rated as especially important for pursuing careers in academic medicine (25, 41). Interestingly, for both sexes, the present study indicated that the pursuit of academic careers was not influenced by the presence of a mentor–mentee relationship. This result is consistent with a previous survey among female surgeons in Switzerland and the finding of Sinclair et al. that academic trainees are less likely to have a surgical mentor (15, 28).

With regard to career satisfaction, mentor–mentee relationships did not have a significant effect. This result is inconsistent with previous studies, where mentoring has been perceived as providing improvement to career satisfaction (12, 42). Similarly, our previous findings also suggest improved career advancement for male surgeons in the presence of a mentor.

This study is primarily limited by methodological factors, as the study involved a survey that relied on subjective information. The main strength of the study is the large sample size; all members of the Swiss Surgical Society working in Switzerland were included in the survey. In addition, the study had a response rate of 58.9%, which is high compared to other studies in the surgical field (43).

In conclusion, mentor–mentee relationships are important for the career advancement of male surgeons. The reason for the lack of an impact on the careers of female surgeons is difficult to judge. The reason may involve a preexisting difference in professional career pursuits between men and women. However, mentoring is not exclusively beneficial for career advancement; mentoring also provides lifelong learning and personal development (37, 44). Thus, mentoring is of crucial significance for both sexes. Although the percentage of surgeons with mentors in Switzerland is high compared to other countries, there remains room for improvement. Specific attention should be paid to the development of more structured mentoring programs.
